# Molecular and morphological clocks for estimating evolutionary divergence times

**DOI:** 10.1186/s12862-021-01798-6

**Published:** 2021-05-12

**Authors:** Jose Barba-Montoya, Qiqing Tao, Sudhir Kumar

**Affiliations:** 1 Institute for Genomics and Evolutionary Medicine, Temple University, Philadelphia, PA 19122 USA; 2 Department of Biology, Temple University, Philadelphia, PA 19122 USA; 3 Center for Excellence in Genome Medicine and Research, King Abdulaziz University, Jeddah, Saudi Arabia

**Keywords:** Species divergence time estimation, Molecular clock, Morphological clock, Fossil calibration, Bayesian inference

## Abstract

**Background:**

Matrices of morphological characters are frequently used for dating species divergence times in systematics. In some studies, morphological and molecular character data from living taxa are combined, whereas others use morphological characters from extinct taxa as well. We investigated whether morphological data produce time estimates that are concordant with molecular data. If true, it will justify the use of morphological characters alongside molecular data in divergence time inference.

**Results:**

We systematically analyzed three empirical datasets from different species groups to test the concordance of species divergence dates inferred using molecular and discrete morphological data from extant taxa as test cases. We found a high correlation between their divergence time estimates, despite a poor linear relationship between branch lengths for morphological and molecular data mapped onto the same phylogeny. This was because node-to-tip distances showed a much higher correlation than branch lengths due to an averaging effect over multiple branches. We found that nodes with a large number of taxa often benefit from such averaging. However, considerable discordance between time estimates from molecules and morphology may still occur as  some intermediate nodes may show large time differences between these two types of data.

**Conclusions:**

Our findings suggest that node- and tip-calibration approaches may be better suited for nodes with many taxa. Nevertheless, we highlight the importance of evaluating the concordance of intrinsic time structure in morphological and molecular data before any dating analysis using combined datasets.

**Supplementary Information:**

The online version contains supplementary material available at 10.1186/s12862-021-01798-6.

## Background

**Fig. 1:**
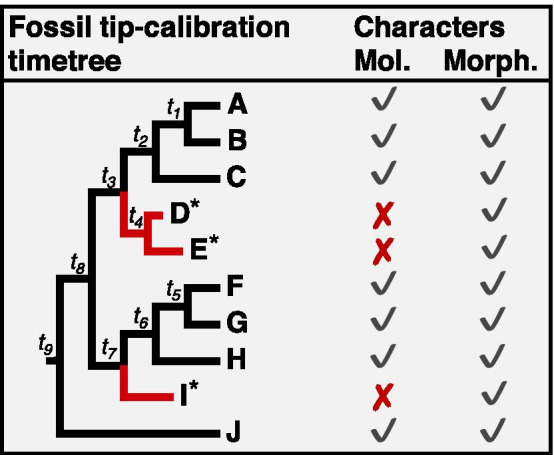
Integration of morphological characters from living and extinct species in a combined analysis with molecular data. The combined matrices contain morphological characters from both fossil (*) and living species, but no molecular data from fossil species. Red branch lengths are created due to the inclusion of extinct taxa and are estimated using non-molecular data. Intuitively, we expect *t*_*3*_, *t*_*4*_, and *t*_*7*_ to be estimated well if the non-molecular data has a strong time structure. The inclusion of non-molecular data will only benefit if the time structure is concordant between molecular and non-molecular datasets. However, we emphasize the importance of using morphological data to infer the phylogenetic position of extinct taxa in the tree to determine which internal nodes are to be calibrated

There is a growing interest in using morphological data to infer species divergence times in systematics by using it in morphological clock analyses (e.g., [1–8]) or combining it with molecular data in total-evidence dating approaches (e.g., [9–14]). In total-evidence dating, morphological characters from dated fossil species and extant species are analyzed along with molecular data under character-specific models of morphological and molecular evolution (e.g., [10, 15]). Tip-calibrations are typically used as constraints on the timing of lineage divergence by including dated fossil species in the relaxed clock analyses [16] (Fig. 1). Moreover, in the fossilized birth–death (FBD) process [17, 18], the fossil species' age provides the calibration information that translates the morphological distances into absolute times and rates, which are propagated to the other nodes on the phylogeny.

Nevertheless, utilizing morphological characters in a dating analysis is complicated [11, 16, 19–23]. Morphological traits are driven by natural selection and adaptation, may experience convergent evolution, and rarely evolve in a clock-like fashion [24, 25]. Furthermore, the sampling of morphological characters is generally focused on taxonomically diagnostic traits. These traits are shared by multiple lineages, with a paucity of autapomorphies and invariant characters, impacting time estimates [22]. Morphological datasets can show an extensive difference in evolutionary rates among branches in the phylogeny, and generally, relatively smaller numbers of morphological characters are sampled compared to phylogenomic datasets [21].

Comparisons of tip-calibration methods applying different FBD models/priors (e.g., [5, 11, 18, 19]) have reported sharp conflict between molecular, and morphological datasets under standard stochastic models causes total-evidence dating to produce older estimates when using inadequate models or vague FBD priors. Furthermore, time estimates are affected by different calibration approaches that lead to different divergence time estimates. The primary source of the problem remains unclear, which may be investigated by examining whether there is significant information in the morphological data to estimate divergence times (time structure). It is also important to evaluate whether the time structure in morphological data is concordant with the time structure in the molecular data. Such concordance will greatly enhance the utility of total-evidence methods in which morphological characters from extinct taxa are combined [10, 12, 13]. It also has implications for FBD dating approaches when molecular and morphological data are jointly used [17] (Fig. 1).

We have compared the intrinsic time structure offered by morphological and molecular data with and without internal node calibrations to avoid confounding the time structure introduced by calibrations. The concordance of time structure between morphological and molecular will be enhanced by the presence of calibrations, which has been the focus of tests by others (e.g., [5, 11, 18, 19]). Figure 1 illustrates how the phylogenetic position of a fossil species (D*, E*, or I*) and the time duration of the branch connecting it to the extant tree are determined based on morphological evidence alone in fossil tip-calibration and FBD dating approaches. The most basic requirement for reliably estimating *t*_*4*_ and *t*_*7*_ is that both morphology and molecules produce concordant time trees. If true, the extrapolation applied in estimating *t*_*4*_ and *t*_*7*_ is appropriate because the molecular data are missing for extinct species. One way to evaluate this hypothesis is to assess node ages' concordance using the living species' data. So, we analyzed three empirical datasets from different species groups, Hemiptera (true bugs), Hymenoptera (ants, bees, sawflies, wasps), and Spermatophyta (seed plants). Moreover, we evaluated the impact of incorporating morphological characters into a joint analysis with molecular on divergence time estimates.

At the outset, we note that morphological information is often used to build evolutionary trees or place fossil taxa in the phylogeny, which usually requires a few diagnostic characters. However, this practice does not generally utilize any information on rates of evolution. Therefore, examining the concordance of intrinsic time structure in morphological and molecular data is independently useful. Its presence is expected to produce better time estimates for the nodes created by the fossil taxa and all other nodes in the molecular tree in the combined fossil and extant taxa analysis.

## Results

### Molecular versus morphological maximum likelihood branch lengths

**Fig. 2:**
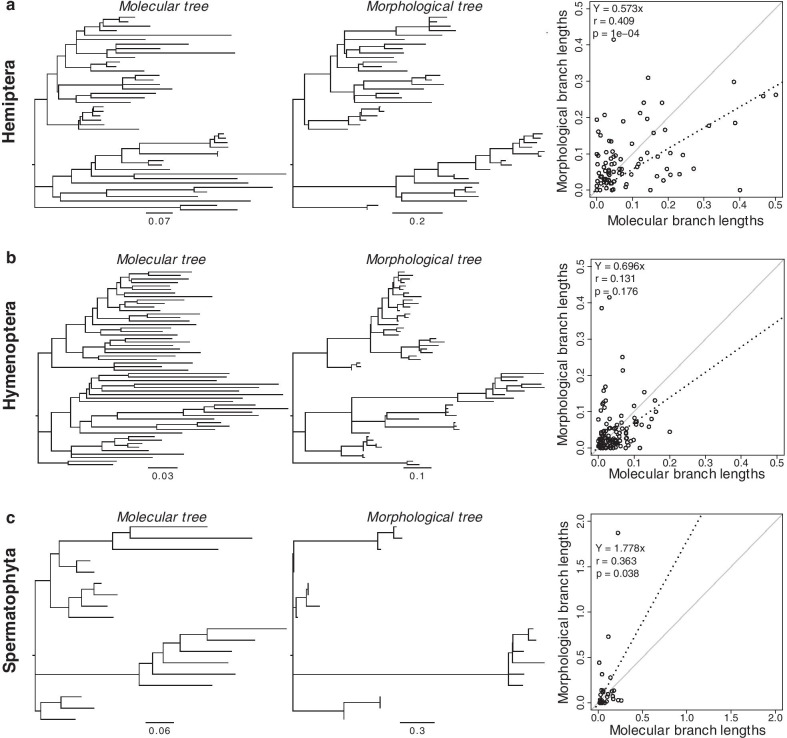
Branch lengths and substitution model parameters were optimized on the same topology for both molecular and morphological data only for **a** Hemiptera, **b** Hymenoptera, and **c** Spermatophyta. The scatterplots to the trees' right show the linear relationship between the branch lengths obtained from molecules versus morphology. The slope, correlation coefficient  (*r*) and p-value are shown. The black dashed line represents the best-fit linear regression through the origin. The solid grey line represents equality between estimates

The maximum likelihood (ML) estimates of branch lengths for the same tree topology were obtained separately for molecular and morphological data. The three datasets, Hemiptera, Hymenoptera, and Spermatophyta, showed a limited correlation and high variation between the molecular and morphological branch lengths (*r* = 0.409, 0.131, and 0.363 respectively; Fig. 2a–c). All three datasets showed rather short terminal morphology-based branch lengths than the molecular data, including several terminal branches of zero length. Overall, the morphological trees had shorter terminal branch lengths as compared to molecular phylogenies. The morphological subsets might not have sampled autapomorphies as much as internal branches' changes, generating artefactually truncated terminal branches for the morphological tree [26]. To examine the effect of analyzing morphological data consisting only of phylogenetically informative characters, we tested for proportionality in internal branch lengths only by excluding terminal branches. The correlations improved for Hemiptera (*r* = 0.529) and Hymenoptera (*r* = 0.622). Spermatophyta showed a weak opposite trend (*r* = 0.028), suggesting discordance on intermediate and deep branches (Additional file 1: Fig. S1). We also tested proportionality in branch lengths by using a likelihood ratio test for nested models as we compared models with proportionate (linked) and unlinked branch lengths. For all three datasets (Hemiptera, Hymenoptera, and Spermatophyta), unlinked models had significantly higher log-likelihoods (Table 1).

**Table 1:** Proportionality in morphological and molecular ML branch lengths

Dataset	Linked branch lengths		Unlinked branch lengths		2∆*l*	p-value
	Parameters	Log-likelihood (*l*)	Parameters	Log-likelihood (*l*)		
Hemiptera	137	-37362.8	222	-**37126.8**	471.9	< 0.001
Hymenoptera	144	-69956.0	253	-**69498.0**	916.1	< 0.001
Spermatophyta	95	-164470.3	128	-**164345.1**	250.4	< 0.001

The model with the highest likelihood in each dataset is shown in bold type

### Assessing molecular and morphological divergence time estimates

#### Analyses without internal calibrations

**Fig. 3:**
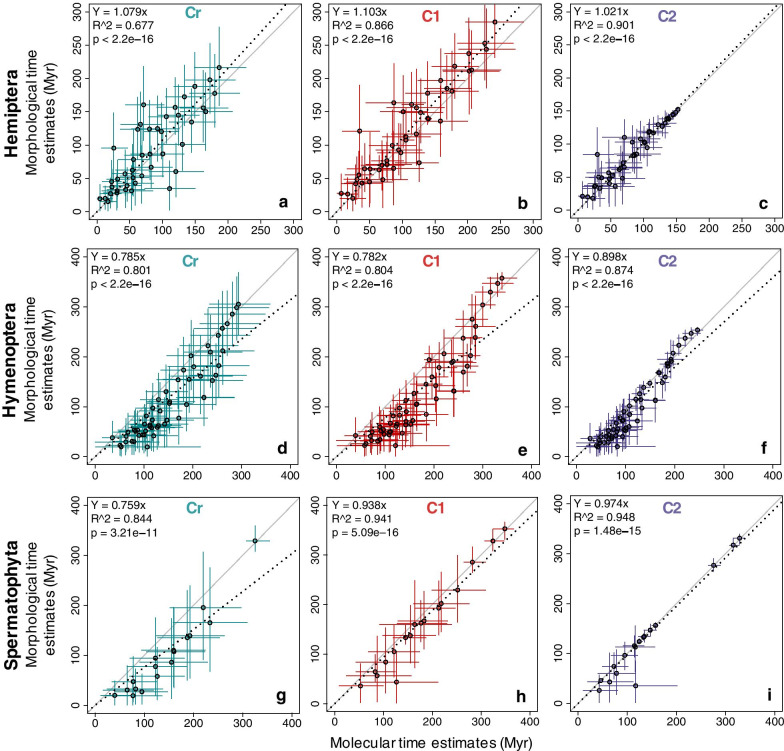
The posterior mean times (empty black dots) and 95% HPD-CIs under calibration strategies Cr (green lines), C1 (red lines), and C2 (purple lines) for the molecular subsets are plotted against the morphological from Hemiptera, Hymenoptera, and Spermatophyta datasets. The slope, coefficient of determination (*R*^2^) for the linear regression through the origin, and p-values are shown. The black dashed line represents the best-fit linear regression through the origin. The solid grey line represents equality between estimates

**Fig. 4:**
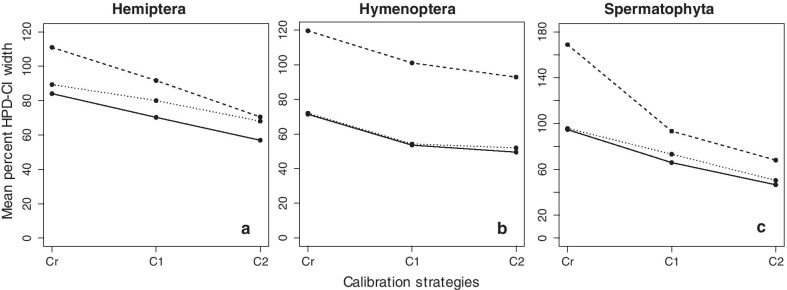
Mean relative width of 95% HPD-CIs (HPD-CI width/time) generated under calibration strategies Cr, C1, and C2 for molecular (dotted lines), morphological (dashed lines), and combined (solid line) subsets from Hemiptera (**a**), Hymenoptera (**b**) and Spermatophyta (**c**) datasets

We first compared time estimates applying only a root calibration (strategy Cr), which is critical to learn about the intrinsic time structure in the data. In the Hemiptera dataset, the correlation between the time estimates obtained from molecules and morphology was significant (*R*^2^ = 0.677; Fig. 3a), which was in contrast to the pattern observed for branch lengths (Fig. 2a). Overall, 72% of morphological node times fell within the 95% highest posterior density credibility intervals (HPD-CIs) for molecular node times. Some of the nodes with significant differences are evident in Fig. 3a and Additional file 1: Fig. S2A (nodes 56, 57, 58, 59, 60, 65, 67, 72, 73, 77, and 79; numbered as in Fig. 8a). These nodes are connected to branches that showed extreme disagreement between morphological and molecular data. We found that morphological data produced wider HPD-CIs than molecular data, the mean of %HPD-CIs (HPD-CI width/time) was 111% and 89%, respectively (Fig. 4a).

In the Hymenoptera dataset, the correlation between the time estimates obtained from molecules and morphology was highly significant (*R*^2^ = 0.801), but so was the variation (Fig. 3d). Morphological data generally produced younger estimates than molecular data, but node times were slightly older than molecular ages for a few nodes (Fig. 3d, Additional file 1: S3A). Nodes that show a large difference in Fig. 3d and Additional file 1: Fig. S3A (nodes 57, 58, 65, 68, 72, 73, 74, 75, 76, 77, 78, 84, 85, and 90; numbered as in Fig. 8b) are related to highly disproportional branches between morphological and molecular data. Only 62% of morphological node times fell within the HPD-CIs for molecular time estimates (Additional file 1: Fig. S3A). Morphological data produced wider HPD-CIs than molecular data. The mean of the %HPD-CIs was 120% and 72%, respectively (Fig. 4b).

In the Spermatophyta dataset, the correlation between the time estimates obtained from molecules and morphology was again high (*R*^2^ = 0.844), but the linear relationship was much tighter (Fig. 3g). This is interesting because the branch lengths showed very little correspondence (see Fig. 2c). For most of the nodes in the timetree, morphological data produced younger estimates than molecular. A likely explanation for younger morphological time estimates than the molecular time estimates is that terminal branches are short for morphological data due to the exclusion of singleton changes (autapomorphy) on terminal branches that result in underestimating times near the tips of the timetree. The nodes with the most significant differences (nodes 20, 21, 28, 29, and 35; numbered as in Fig. 8c) are related to highly discordant morphological and molecular branches (Fig. 3g, Additional file 1: Fig. S4A). 88% of morphological node times fell within the HPD-CIs for molecular time estimates (Additional file 1: Fig. S4A). Morphological data produced wider HPD-CIs than molecular data. The mean of the %HPD-CIs was 169% and 96%, respectively (Fig. 4c).

In the three datasets analyzed, the uncertainty of morphological divergence time estimates was higher than that from molecular data (Fig. 4a–c). The reason appears to be because morphological characters evolve at much more variable rates than molecules and are sampled in relatively smaller numbers than molecular characters, increasing the uncertainty in posterior time estimates and, therefore, the HPD-CI widths of morphological time estimates.

#### Analyses with multiple calibrations

We expected the above results to improve with the addition of internal calibrations. So, we used multiple calibration constraints but made them diffuse in our first analyses, reflecting agnosticism on the fossil ages, which is considered a more realistic scenario (strategy C1). Indeed, in the Hemiptera dataset, the correlation between the time estimates obtained from molecules and morphology became higher under this calibration strategy (*R*^2^ = 0.866; Fig. 3b). It is worth mentioning that, although differences were still found between age estimates from morphology and molecules, on average, timescales were similar (Fig. 3b, slope = 1.103). 85% of morphological node times fell within the HPD-CIs for molecular time estimates (Additional file 1: Fig. S2B). Morphological and molecular data produced similar HPD-CIs. The mean of the %HPD-CIs from morphological data was 92% and 80% from molecular data (Fig. 4a).

In the Hymenoptera dataset, the correlation between the time estimates obtained from molecules and morphology remained similar (*R*^2^ = 0.804; Fig. 3e) than in strategy Cr. Morphological data produced younger estimates than molecular, except for a few node times, which were slightly older than molecular ages (Fig. 3e, Additional file 1: Fig. S3B). 42% of morphological node times fell within the HPD-CIs for molecular time estimates (Additional file 1: Fig. S3B). Morphological data produced wider HPD-CIs than molecular data. The mean of the %HPD-CIs from morphological data was 101% and 54% from molecular data (Fig. 4b). In the Spermatophyta dataset, the correlation between the time estimates obtained from molecules and morphology was very high (*R*^2^ = 0.941; Fig. 3h). Morphological data produced nearly identical time estimates to molecular dates, except for node 28 that was very young compared to molecular dates (Fig. 3h, Additional file 1: Fig. S4B). 94% of morphological node times fell within the HPD-CIs for molecular time estimates (Additional file 1: Fig. S4B). Morphological data produced wider HPD-CIs than molecular data. The mean of the %HPD-CIs from morphological data was 93% and 73% from molecular (Fig. 4c). The use of multiple internal calibrations reduced the uncertainty of divergence time estimates for both morphological and molecular data in the three datasets (Hemiptera, Hymenoptera, and Spermatophyta), producing narrower HPD-CIs than in strategy Cr.

#### Analyses with multiple narrow calibrations

In many studies, however, investigators apply narrower calibration densities. So, we applied narrow calibration constraints in strategy C2, reflecting a prior assumption that fossil ages are relatively close to the "true" age of the corresponding lineage. We thus expected an even greater concordance between morphological and molecular estimates of the above results when we use multiple narrow calibrations. In effect, in the Hemiptera dataset, the concordance of dates between morphological and molecular data became higher (*R*^2^ = 0.901; Fig. 3c, Additional file 1: Fig. S2C). The differences among molecular and morphological time estimates were the smallest for calibration strategy C2, showing a linear slope close to 1. Although differences were found between age estimates from morphology and molecules, on average, timescales were similar. Under strategy C1, 85% of morphological node times fell within the HPD-CIs for molecular time estimates, while this proportion increased to 96% under strategy C2 (Fig. 3b, c, Additional file 1: Fig. S2). Morphological and molecular data produced similar HPD-CIs. The mean of the %HPD-CIs from morphological data was 70% and 68% (Fig. 4a).

In the Hymenoptera dataset, the concordance of dates also improved (*R*^2^ = 0.874; Fig. 3f, Additional file 1: Fig. S3C). As under strategy C1, morphological data produced mostly younger estimates than molecular (with only a few exceptions; Fig. 3f). The differences among molecular and morphological time estimates were the smallest for calibration strategy C2, showing a linear slope close to 0.9, and 69% of morphological node times fell within the HPD-CIs for molecular time estimates (Fig. 3f, Additional file 1: Fig. S3C). Morphological data produced wider HPD-CIs than molecular data. The mean of the %HPD-CIs from morphological data was 93% and from molecular data was 52% (Fig. 4b).

In the Spermatophyta dataset, the concordance of dates from morphological and molecular subsets remained high (*R*^2^ = 0.948; Fig. 3i). As under strategy C1, morphological data produced nearly identical time estimates to molecular data, except for node 28, which was very young compared to molecular dates (Fig. 3i, Additional file 1: Fig. S4C). The differences among molecular and morphological time estimates were the smallest for calibration strategy C2, showing a linear slope close to 1. 94% of morphological node times fell within the HPD-CIs for molecular time estimates (Fig. 3i, Additional file 1: Fig. S4C). Morphological data produced wider HPD-CIs than molecular data. The mean of the %HPD-CIs from morphological data was 68% and 50% from molecular data (Fig. 4c). The strong similarity between morphological and molecular time estimates under this calibration strategy with narrow probability densities occurs because nodes were constrained within a restricted period forcing the time prior and posterior estimates into an agreement so that both molecular and morphological data played a minor role in inferring divergence times [27–29]. Moreover, precise calibrations reduced the uncertainty of divergence time estimates producing narrow HPD-CIs and concordance between morphological and molecular data.

#### Assessing divergence time estimates from combined datasets

**Fig. 5:**
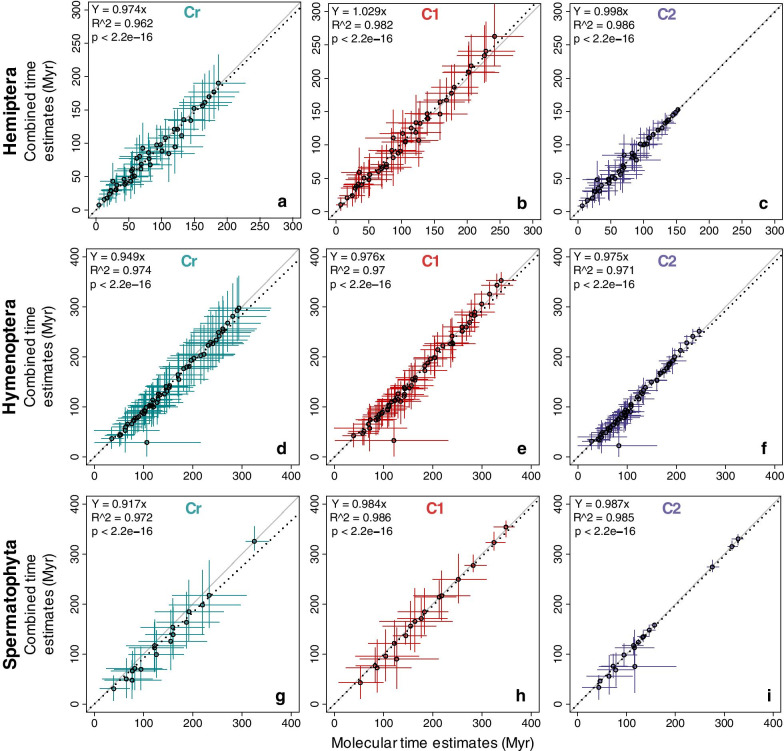
The posterior mean times (empty black dots) and 95% HPD-CIs under calibration strategies Cr (green lines), C1 (red lines), and C2 (purple lines) for the molecular subsets are plotted against the combined from Hemiptera, Hymenoptera, and Spermatophyta dataset using unlinked clock models. The slope, coefficient of determination (*R*^2^) for the linear regression through the origin, and p-values are shown. The black dashed line represents the best-fit linear regression through the origin. The solid grey line represents equality between estimates

Divergence time estimates from the Hemiptera combined subset were very similar to molecular estimates under the three calibration strategies. However, morphological data produced slightly younger estimates than molecular data under calibration strategies Cr and C1 (Fig. 5a, b). However, under both calibration strategies, only a few node times deviated from a 1:1 linear trend (Fig. 5a, b). The mean of the %HPD-CIs from the combined subset was 84% under strategy Cr, 70% under C1, and 57% under C2 (Fig. 4a). Under the three calibration strategies, 100% of combined node times fell within the HPD-CIs for molecular time estimates (Fig. 5a–c, Additional file 1: Fig. S2). Time estimates from the Hymenoptera combined subset were almost identical to molecular estimates under the three calibration strategies, and only a few node times deviated from a 1:1 linear trend. Mainly, node 58 was consistently older for the molecular subset under the three strategies. A possible reason is that the ancestral and descendant branches of node 58 have extremely different lengths in the molecular and morphological trees (Fig. 2b). This pattern persisted in the posterior time estimates. Under the three calibration strategies, 100% of combined node times fell within the HPD-CIs for molecular time estimates (Fig. 5d–f, Additional file 1: Fig. S3). The mean of the %HPD-CIs from the combined subset was 71% under strategy Cr, 54% under C1, and 50% under strategy C2 (Fig. 4b). Divergence time estimates from the Spermatophyta combined subset were nearly equal to molecular estimates under all calibration strategies, none of the nodes deviated from a 1:1 linear trend (Fig. 5g–i, Additional file 1: Fig. S4). The mean of the %HPD-CIs from the combined subset was 95% under strategy Cr, 66% under C1, and 47% under C2 (Fig. 4c). Overall, combining morphological and molecular datasets had little effect on divergence time estimates. The strong similarity between combined and molecular time estimates appears to be partly due to the relatively small morphological datasets and their low information content due to variable rates [16].

#### Bayes factor calculation for clock model selection

We tested for proportionality in time estimates by using the Bayes factor to compare models with linked and unlinked relaxed-clock models [30]. Two analyses were performed, one using a single clock for the entire morphological and molecular dataset (linked clocks) and the other with unlinked clocks for morphological and molecular data [26]. To evaluate the two-clock model's performance, we used marginal likelihood calculation to estimate Bayes factors and posterior model probabilities. We used the stepping-stone method to obtain accurate model likelihoods [31].

The model likelihoods are presented in Table 2. Model likelihoods show a large difference between the two models for three datasets (Hemiptera, Hymenoptera, and Spermatophyta). The marginal likelihood estimates for the single clock model is worse than the unlinked model in all cases. Thus, the stepping-stone marginal likelihood indicates strong evidence in favor of unlinked clock models for morphological and molecular data. However, time estimates between the two clock models were almost identical (Fig. 4, Additional file 1: Fig. S5), even though a Bayes factor test very strongly supported the unlinked clock model,

**Table 2:** Bayes factor (BF) calculation for clock-model selection

Dataset	Linked clock model marginal likelihood	Unlinked clock model marginal likelihood	Stepping-stone BF
Hemiptera Cr	-37081.9	-**36948.1**	-133.7
Hymenoptera Cr	-69571.4	-**69288.3**	-232.1
Spermatophyta Cr	-164373.8	-**164309.4**	-64.4

The model with the highest posterior probability in each dataset is shown in bold type

## Discussion

While many investigators have compared node ages estimated from morphological and molecular data, they have generally used multiple calibrations in their analyses (e.g., [1, 5, 8]). They often report that both data sources produce comparable time estimates, but some morphological estimates have been older than their molecular counterparts. Others have compared node age estimates obtained from total-evidence dating involving tip dates with those employing node-calibrations in dating [5, 10, 13, 32–34]. They have reported that estimates from the total-evidence analysis are less sensitive to prior assumptions and tend to have smaller confidence/credibility intervals. However, total-evidence analysis tends to produce older node age estimates, with the difference likely due to differences in the time-prior used for node-calibration and total-evidence dating.

In this study, we explored the relationship of ML estimates from the molecular and morphological branch lengths for the same phylogeny (Fig. 2a–c). We observed that morphological evolution along the tree was generally uncoupled from molecular evolution, as the branch length correlation was low and the dispersion high in the three datasets analyzed (Hemiptera, Hymenoptera, and Spermatophyta). Morphological phylogenies also differ from molecular phylogenies in having shorter terminal branches, a pattern that was common to all three datasets. The low correlation suggests that morphological characters evolve at much more variable rates than molecules, and the shorter terminal branches are likely to be due to ascertainment bias. We expected these disagreements between molecular and morphological branch lengths to have a significant detrimental impact on the efficacy of dating analysis.

**Fig. 6:**
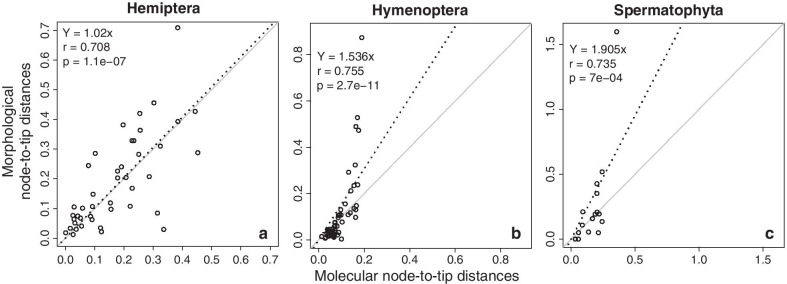
The linear relationship between node-to-tip distances from molecules alone versus morphology for **a** Hemiptera, **b** Hymenoptera, and **c** Spermatophyta. The black dashed line represents the best-fit linear regression through the origin. The slope, correlation coefficient (*r*) and p-values are shown. The black dashed line represents the best-fit linear regression through the origin. The solid grey line represents equality between estimates

However, the observed concordance between divergence times produced by these two types of data seems to be much more than that anticipated based on comparing individual branch lengths. One fundamental reason for this observation is that relative node-to-tip distances in morphological and molecular phylogenies have a much better correlation than the branch lengths (Fig. 6). The node-to-tip distance estimate for a node is the sum of branch lengths of all paths from this node to all descendent tips divided by the total number of descendant tips. In the Hemiptera dataset, the correlation between node-to-tip distances (*r* = 0.708) was much higher than that for the branch lengths (*r* = 0.409), although the relationship was still noisy (Fig. 6a). In the Hymenoptera and the Spermatophyta datasets, the correlation between node-to-tip distances was also high (*r* = 0.755 and 0.735 respectively) compared to the branch lengths (*r* = 0.131, and 0.363, respectively). The dispersion was lower for each dataset than that for branch lengths (Fig. 6b, c).

**Fig. 7:**
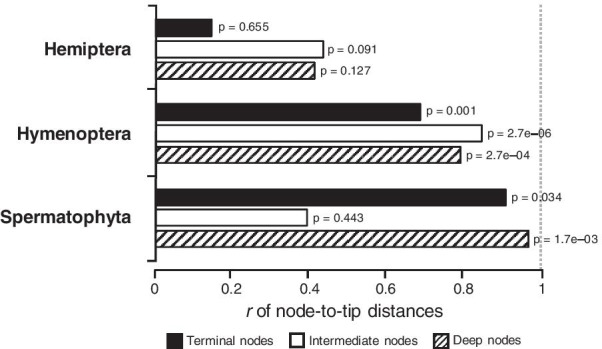
Linear relationships of molecular and morphological estimates of node-to-tip distances for nodes that locate at the terminal (solid), intermediate (open), and deep (hatch) divergence regions for Hemiptera, Hymenoptera, and Spermatophyta. An *r* of one indicates a perfect linear relationship between molecular and morphological estimates, marked as the gray dashed line. P-values are shown next to the bars

Therefore, a better relationship between node-to-tip distances from morphological and molecular data is a primary reason for the concordance between divergence times produced by these two types of data. The averaging of path lengths to multiple descendants in calculating node-to-tip distances is likely creating the time structure in both molecular and morphological phylogenies, as they have the same branching pattern. If so, we would expect this benefit to be weaker for nodes with fewer descendants because there are fewer evolutionary paths to average over. This effect was observed in the Hemiptera and Hymenoptera datasets; terminal nodes showed weaker node-to-tip distance relationships reflected in their significant deviation from the linear trend (Fig. 7). The terminal nodes represent the split of one lineage to form two living taxa, for which the *r* was similar  in the node-to-tip comparison  to that for the branch lengths.

The linear relationships of molecular and morphological node-to-tip distance estimates were higher for intermediate divergence nodes (Fig. 7). These nodes had at least three descendants, excluding the root and nodes representing the set of deep divergence nodes. Then, the linear relationships of molecular and morphological node-to-tip distance estimates of the deepest nodes were also higher than for terminal nodes. This suggests that for the Hemiptera and Hymenoptera datasets, tip-calibration methods might be better suited for nodes with many taxa. However, the linear relationships of molecular and morphological node-to-tip distance estimates of the intermediate nodes were the lowest in the Spermatophyta dataset, indicating discordance of estimated dates for intermediate nodes. Therefore, tip-calibration methods might be better suited for deep and shallow nodes for this dataset (Fig. 7). Based on our results, it seems possible that the duration of the branches connecting fossils to the extant tree, which are determined based on morphological evidence, may not be wrongly inferred for deepest and intermediate nodes in tip-calibration and FBD dating approaches (see also, Püschel et al. [20]). The discordance observed is because the Bayesian dating analysis tends to be too sensitive to BD (birth–death) process used to specify the time prior without constraints on interior nodes [24].

**Fig. 8:**
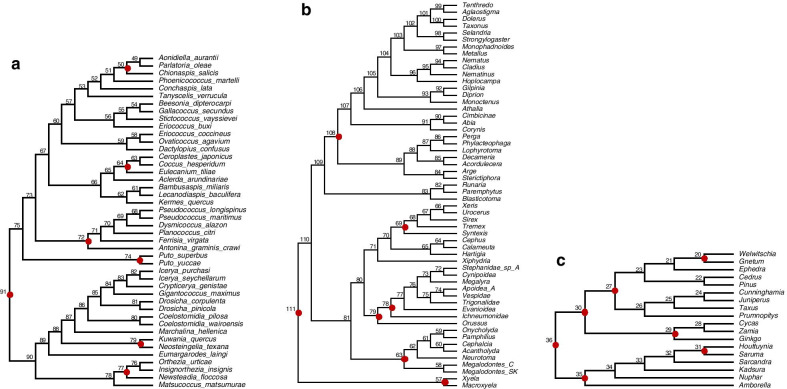
Phylogenies for **a** 44 Hemiptera species, **b** 56 Hymenoptera species, and **c** 18 Spermatophyta species. Closed red dots indicate calibration nodes

Moreover, the high concordance of relative time estimates between morphological and molecular data when multiple internal calibrations are applied, and the extreme similarity of time estimates between combined and molecular data, justify the use of morphological data in node-, tip-calibration and FBD dating approach. This kind of analysis is especially useful for total-evidence approaches because they are likely impacted by the relationship between morphological and molecular estimates, in contrast to node-dating approaches that do not use morphological data. Nevertheless, the concordance of time estimates between datasets needs to be carefully examined. As we showed, discordance on time structure can only be corrected by calibrations, but some nodes remain different (e.g., Hemiptera nodes 58 and 59; Hymenoptera nodes 58 73, 74, 75, and 85; Spermatophyta node 28. Additional file 1: Figs. S2, S3, S4; nodes are numbered as in Fig. 8a–c).

We examined if the high correlation between morphological and molecular time estimates could arise due to using the same topology and speciation tree prior in the Bayesian analysis (suggested by S. Guindon). We generated time estimates using a uniform BD branching process in MCMCTree [35], conditioning on the topology and root node calibration. Neither molecular nor morphological data were used. The correlation between the resulting times was determined to evaluate the time structure imposed by the tree prior when the tree topology is fixed. The correlation between node times generated using a uniform BD branching process and time estimates obtained from molecular and morphological data was high for the three datasets (*R*^2^ = 0.733, 0.5 and 0.84 for molecular subsets and *R*^2^ = 0.841, 0.726 and 0.722 for morphological subsets), rivaling the correlation between molecular and morphological time estimates (*R*^2^ = 0.677, 0.801, 0.844 for Hemiptera, Hymenoptera and Spermatophyta, respectively). While we expected some correlation because of the same time prior and branching process, which results in drawing times from the same probability distribution of times for each node in the phylogeny [24], the magnitude of correlation observed was surprising. Therefore, the species phylogeny and the use of the same tree-prior would introduce similar time structures for different data types in their joint Bayesian analysis. In the future, we plan to examine how this correlation of time structure affects the accuracy of Bayesian node age estimates, as compared to the performance non-Bayesian methods that estimate times based primarily on branch lengths without using any speciation tree-priors (Tao et al. 2020).

## Conclusions

Overall, our study allows us to conclude that (1) relative ML branch lengths between morphological and molecular characters were very different, but relative node-to-tip distances were considerably more concordant, suggesting a much more concordant time structure in the morphological and molecular dataset than that captured in the comparison of branch lengths. (2) When no internal calibrations were applied, morphological and molecular clock produced time estimates with a high correlation, which may be caused by the same speciation tree prior applied in the joint consideration of molecular and morphological data in the Bayesian analyses. The concordance between time estimates was improved by applying multiple and/or narrow internal calibrations. (3) The combination of molecular and morphological data generally resulted in time estimates nearly identical to the ones from molecular data alone. Our study allows us to conclude that although there is a concordant time structure in morphological and molecular data, the interpretation of fossil ages is determinant for the agreement of time estimates from morphological and molecular clock analyses. We emphasize the importance of evaluating the concordance of time structure in morphological and molecular data before any dating analysis using combined datasets.

## Methods

### Taxon sampling, molecular and morphological data, and tree topologies

For comparison between results from molecules and morphology, we composed three datasets consisting of extant species, for which both molecular and morphological data were available. Thus, extinct taxa were not included in our matrices. First, a dataset of 174 discrete morphological characters and 3731 molecular characters (base pairs—bp) from 44 Hemiptera species was obtained from Vea and Grimaldi (2016) [32]. Second, a dataset of 353 discrete morphological characters and 5096 molecular characters (bp) from 55 Hymenoptera species was taken from Ronquist et al. (2012) [10]. Third, a dataset of 121 discrete morphological characters and 19,870 molecular characters (bp) from 18 Spermatophyta species was obtained from Doyle (2006) [36] and Morris et al. (2018) [37], respectively. Three rooted trees (Fig. 8) were constructed based on the literature: one for Hemiptera species [32], one for Hymenoptera species [10], and one for Spermatophyta species [37]. The three topologies from Fig. 8 were fixed in all analyses to ensure consistent calibrations and avoid the confounding effects of alternative phylogenies and calibrations. Moreover, the use of a phylogeny reliably inferred as a fixed topology for dating is a common practice (e.g., [7, 37–40]).

### Fossil calibrations and calibration strategies

Fossil calibrations are the foremost source of information for translating the distances between molecular/morphological sequences into estimates of absolute times and absolute rates in clock dating analysis. Thus, the calibrations' quality is expected to impact divergence time estimates significantly, even if a large amount of sequence data is available. Therefore, we employed three fossil calibration strategies simulating different quality of calibrations based on fossil evidence. Fossil calibrations were employed following justifications from the original studies (Hemiptera [32], Hymenoptera [10], Spermatophyta [27]; Fig. 8 and Table 3). For the three strategies, node calibrations were specified using a uniform distribution from fossil minimum-ages (Table 3).

**Table 3:** Summary of fossil calibrations and calibration strategies used in this study in million years before the present

Dataset	Node	Clade	Minimum divergence time (Ma)	Calibration strategy Cr	Calibration strategy C1	Calibration strategy C2
Hemiptera	91 (root)	Coccomorpha	140 (†*Eomatsucoccus casei*)	U(140,340)	U(140,340)	U(140,354)
Hemiptera	79	Kuwaniidae	45 (†*Hoffeinsia foldi*)	N/A	U(45,155)	U(45,50)
Hemiptera	77	Ortheziidae	135 (†*Cretorhezia hammanaica*)	N/A	U(135,145)	U(135,149)
Hemiptera	74	Putoidae	45 (†*Puto* sp.)	N/A	U(45,155)	U(45,50)
Hemiptera	72	Pseudococcidae	135 (†*Williamsicoccus megalops*)	N/A	U(135,145)	U(135,149)
Hemiptera	64	Coccidae	98 (†*Rosahendersonia prisca*)	N/A	U(98,122)	U(98,108)
Hemiptera	50	Diaspididae	50 (†*Normarkicoccus cambayae*)	N/A	U(50,150)	U(50,55)
Hymenoptera	111 (root)	Hymenoptera	235 (†*Triassoxyela, Asioxye*la)	U(235,369)	U(235,369)	U(235,259)
Hymenoptera	108	Tenthredinoidea	140 (†*Palaeathalia*)	N/A	U(140,328)	U(140,154)
Hymenoptera	79	Vespina	180 (†*Brigittepteris*)	N/A	U(180,290)	U(180,198)
Hymenoptera	78	Apocrita	176 (†*Cleistogaster*)	N/A	U(176,294)	U(176,194)
Hymenoptera	69	Siricoidea	161 (†*Aulisca*)	N/A	U(161,309)	U(161,177
Hymenoptera	63	Pamphiloidea	161 (†*Aulidontes, Pamphilidae* sp.)	N/A	U(161,309)	U(161,177)
Hymenoptera	57	Xylidae	180 (†*Eoxyela*)	N/A	U(180,298)	U(180,198)
Spermatophyta	36 (root)	Spermatophytes	308.14 (†*Cordaites iowensis*)	U(308,366)	U(308,366)	U(308,339)
Spermatophyta	35	Angiosperms	125.9 (tricolpate pollen)	N/A	U(126,247)	U(126,139)
Spermatophyta	31	Stem-Saururus	44.3 (†*Saururus tuckerae*)	N/A	U(44,247)	U(44,48)
Spermatophyta	30	Acrogymnosperms	308.14 (†*Cordaties iowensis*)	N/A	U(308,366)	U(308,339)
Spermatophyta	29	Gingko-Cycas	264.7 (†*Crossozamia*)	N/A	U(265,366)	U(265,282)
Spermatophyta	27	Conifers	147 (†*Rissikia media*)	N/A	U(147,312)	U(147,162)
Spermatophyta	20	Gnetales	119.6 (†*Eoantha zherkihinii*)	N/A	U(120,312)	U(120,132)

Nodes are numbered as in Fig. 8. Fossil taxa are indicated by a dagger (†) before their names. N/A, not applicable

#### Calibration strategy Cr

Only one calibration on the root was specified (no internal calibrations), assigning a uniform distribution borrowed from calibration strategy C1 as described below (Table 3). This strategy reflects a poor fossil record and the lack of fossil evidence for the internal nodes. However, it has the advantage of being the baseline for examining the impact of using calibration constraints on divergence time estimates.

#### Calibration strategy C1

Calibrations were specified using a uniform distribution U(*t*_L_, *t*_U_), where *t*_L_ is the minimum age bound and *t*_U_ the maximum age bound, with mean ${t}_{\mathrm{M}}= \frac{1}{2} ({t}_{\mathrm{L}}+ {t}_{\mathrm{U}})$. The offset and mean values from the original calibration were used respectively as the minimum bound (*t*_L_) and mean (*t*_M_) for the uniform distribution so that the maximum bound was ${t}_{\mathrm{U}}= \frac{{t}_{\mathrm{M}}}{0.5}- {t}_{\mathrm{L}}$ (Table 3). This strategy reflects agnosticism about the true time of divergence between these bounds, which is considered a more realistic scenario.

#### Calibration strategy C2

Calibrations were also specified using a uniform distribution U(*t*_L_, *t*_U_). The offset value from the original exponential distribution was used as the minimum bound (*t*_L_), and the maximum bound was specified at ${t}_{\mathrm{U}}={t}_{\mathrm{L}}+ \frac{{t}_{\mathrm{L}}}{10}$, which assigns a probability of 10% for the minimum bound (*t*_L_) to be older (Table 3). This strategy reflects a prior belief that the fossil minima are a close approximation of clade age. Such a strategy is only appropriate for calibration nodes within a restricted time, assuming that fossil ages represent the "true" age of the corresponding lineage.

### Modeling morphological and molecular evolution

To model morphological evolution, we used the Mk model [15], with the ascertainment bias set to variable (only variable characters scored), equal state frequencies, and assuming discrete gamma-distributed heterogeneity among sites (Mk + Γ_5_). To model molecular evolution, we used the GTR general time-reversible (GTR) model [41], with discrete gamma-distributed heterogeneity among sites (GTR + Γ_5_). Unlike the model applied for morphological data (Mk + Γ_5_), the GTR + Γ_5_ model relaxes the assumption of equal character frequencies in molecular substitutions. A ML optimization of model parameters and branch lengths for the given topology was performed for both molecular and morphological data separately in MEGAX [42] and RAxML [43], respectively, for the Hemiptera, Hymenoptera, and Spermatophyta datasets to assess the pattern of branches lengths from the morphological and molecular subsets. Tests of proportionality of branch lengths were performed using a likelihood ratio test to compare nested models with either linked or unlinked branch lengths in RAxML-ng [44]. We also calculated the node-to-tip distances using the ML trees. For each node, the node-to-tip distance is the sum of branch lengths of all paths from this node to all descendent tips divided by the total number of descendant tips.

### Bayesian divergence time estimation

In each data analysis (Hemiptera, Hymenoptera, and Spermatophyta), we used one molecular subset, one morphological subset, and one combined (morphological + molecular) subset, and applied three calibration strategies, Cr, C1, and C2. All Bayesian analyses (27 in total) were carried out with the program MrBayes [31]. The sequence likelihood of the morphological and molecular subsets was calculated under the Mk + Γ_5_ and the GTR + Γ_5_ substitution models, respectively. The combined subset was treated as two partitions under separate substitution models (Mk + Γ_5_, and GTR + Γ_5_), with separate substitution-rate parameters assigned and estimated for each partition. Two sets of analyses were performed, using: a single clock for the entire morphological and molecular dataset (linked clocks) or unlinked clocks for morphological and molecular data [26]. The MCMC sampling settings were determined through pilot runs and differed among the datasets. We ran each analysis at least twice and checked for convergence by comparing the posterior mean estimates between runs and plotting the samples' time series traces. We then merged the samples from the runs before summarizing the posterior. Moreover, we ran analyses without sequence data to estimate the effective time prior that informed the resulting marginal priors for the node ages after truncation. We plotted these alongside node age estimates from the posteriors to assess how much the data influences age estimates (Additional file 1: Figs. S2, S3, S4). The prior settings and the MCMC run parameters of each dataset analysis are detailed below. For the Bayes factor test, marginal likelihoods were estimated using stepping-stone sampling based on 50 steps with 490,000 generations (490 samples) within each step.

#### Analysis of Hemiptera dataset

Analyses were performed on three subsets from 44 Hemiptera species, one of 174 morphological characters with 12.15% of missing data; one of 3731 molecular characters (bp) with 47.96% of missing data; and one combined subset (morphological + molecular) of 3905 characters with 46.36% of missing data. The rooted tree topology with seven calibration nodes (Fig. 8a) was fixed in all analyses. Detailed information on the calibrations is given in Table 3. Models of evolution for each subset were implemented, as described earlier. Priors for rates were set as in Vea and Grimaldi (2016) [32]. The *Independent Gamma Rate* (IGR) model, in which the rates of evolution on branches varied independently from a gamma distribution [45], was used as a rate prior. The gamma model is parametrized using two parameters: the mean and variance. The time unit was 100 Myr. The mean was assigned a lognormal hyperprior L.N. (− 6.0605,0.0519), with the mean of exp{− 6.0605 + 0.0519^2^/2} = 0.0023. The variance (*Igrvarpr*) was assigned an exponential hyperprior with a mean of 0.039. Analyses were performed with either linked or unlinked relaxed clock models (i.e., link Igrvar = (all)) or unlink Igrvar = (all)). For each analysis, four MCMC runs were performed, each consisting of 5 × 10^6^ iterations, sampling every 200, with the burn-in set to 10%, resulting in a total of 9 × 10^4^ samples from the four runs, which were used to obtain posterior time estimates.

#### Analysis of Hymenoptera dataset

Analyses were performed on three subsets from 56 Hymenoptera species; one of 353 morphological characters with 19.97% of missing data; one of 5096 molecular characters (bp) with 25.65% of missing data; and one combined subset (morphological + molecular) of 5449 characters with 25.28% of missing data. The rooted tree topology with seven calibration nodes (Fig. 8b) was fixed in all analyses. Detailed information on the calibrations is given in Table 3. Models of evolution for each subset were implemented, as described earlier. Priors for rates were set as in Ronquist et al. (2012) [10] for the three subsets. The IGR model was used to specify prior for rates. The time unit was 100 Myr. The mean of the gamma was assigned a lognormal hyperprior LN(− 7.1, 0.5), with the mean exp{− 7.1 + 0.5^2^/2} = 0.001. The variance of the gamma was assigned an exponential hyperprior with a mean of 0.027. Analyses were performed with either linked or unlinked relaxed clock models (i.e., link Igrvar = (all)) or unlink Igrvar = (all)). Four MCMC runs were performed, each consisting of 5 × 10^6^ iterations, sampling every 200, with the burn-in set to 10%, resulting in a total of 9 × 10^4^ samples from the four runs.

#### Analysis of Spermatophyta dataset

Analyses were performed on three subsets from 18 Spermatophyta species; one of 121 morphological characters with 27.5% of missing data; one of 19,870 molecular characters (bp) with 14.11% of missing data; and one combined subset (morphological + molecular) of 5449 characters with 14.18% of missing data. The rooted tree topology with seven calibration nodes (Fig. 8c) was fixed in all analyses. Detailed information on the calibrations is given in Table 3. Models of evolution for each subset were implemented, as described earlier. Priors for rates were set as in Barba-Montoya et al. (2018) [27] for the three subsets. The IGR model was used to specify prior for rates. The time unit was 100 Myr. The mean of the gamma was assigned a lognormal hyperprior LN (− 2.79, 0.5), with the mean exp{− 2.79 + 0.5^2^/2} = 0.07, and the variance of the gamma is assigned an exponential hyperprior with a mean of 0.1. Analyses were performed with either linked or unlinked relaxed clock models (i.e., link Igrvar = (all)) or unlink Igrvar = (all)). Six MCMC runs were performed, each consisting of 1 × 10^7^ iterations, sampling every 200, with the burn-in set to 10%, resulting in a total of 2.7 × 10^5^ samples from all six runs.

## Supplementary Information

**Additional file 1: Figure S1.** The linear relationship between internal branch lengths obtained from molecules versus morphology (excluding terminal branches) for (A) Hemiptera, (B) Hymenoptera, and (C) Spermatophyta. The slope, correlation coefficient (*r*) and p-values are shown. The black dashed line represents the best-fit linear regression through the origin. The solid grey line represents equality between estimates. **Figure S2.** Calibration densities (dark grey bands), 95% HPD-CIs in the time prior (light grey bands), and posterior (colored lines) for 47 nodes in the Hemiptera timetrees under calibration strategies (A) Cr-green, (B) C1-red, and (C) C2-purple. **Figure S3.** Calibration densities (dark grey bands), 95% HPD-CIs in the time prior (light grey bands), and posterior (colored lines) for 55 nodes in the Hymenoptera timetrees under calibration strategies (A) Cr-green, (B) C1-red, and (C) C2-purple. **Figure S4.** Calibration densities (dark grey bands), 95% HPD-CIs in the time prior (light grey bands), and posterior (colored lines) for 17 nodes in the Spermatophyta timetrees under calibration strategies (A) Cr-green, (B) C1-red, and (C) C2-purple. **Figure S5.** The posterior mean times (empty black dots) and 95% HPD-CIs under calibration strategies Cr (green lines), C1 (red lines), and C2 (purple lines) for the molecular subsets are plotted against the combined from Hemiptera, Hymenoptera, and Spermatophyta datasets using linked clock models.

## Data Availability

The datasets generated and/or analyzed during the current study are available in the figshare repository: https://doi.org/10.6084/m9.figshare.9775730. [46].

## Abbreviations

FBD: Fossilized birth–death
ML: Maximum likelihood
CIs: Credibility intervals
BF: Bayes factor
BD: Birth–death
bp: Base pairs
GTR: General time-reversible
