# Supplementary material for: Molecular and morphological clocks for estimating evolutionary divergence times
Source: BMC Ecol Evol. 2021 May 12;21:83. doi: 10.1186/s12862-021-01798-6 (PMC8117668; doi:10.1186/s12862-021-01798-6)
Supplement: Supplementary file 1 — Additional file 1: Figure S1. The linear relationship between internal branch lengths obtained from molecules versus morphology (excluding terminal branches) for (A) Hemiptera, (B) Hymenoptera, and (C) Spermatophyta. The slope, correlation coefficient (r) and p-values are shown. The black dashed line represents the best-fit linear regression through the origin. The solid grey line represents equality between estimates. Figure S2. Calibration densities (dark grey bands), 95% HPD-CIs in the time prior (light grey bands), and posterior (colored lines) for 47 nodes in the Hemiptera timetrees under calibration strategies (A) Cr-green, (B) C1-red, and (C) C2-purple. Figure S3. Calibration densities (dark grey bands), 95% HPD-CIs in the time prior (light grey bands), and posterior (colored lines) for 55 nodes in the Hymenoptera timetrees under calibration strategies (A) Cr-green, (B) C1-red, and (C) C2-purple. Figure S4. Calibration densities (dark grey bands), 95% HPD-CIs in the time prior (light grey bands), and posterior (colored lines) for 17 nodes in the Spermatophyta timetrees under calibration strategies (A) Cr-green, (B) C1-red, and (C) C2-purple. Figure S5. The posterior mean times (empty black dots) and 95% HPD-CIs under calibration strategies Cr (green lines), C1 (red lines), and C2 (purple lines) for the molecular subsets are plotted against the combined from Hemiptera, Hymenoptera, and Spermatophyta datasets using linked clock models. [file 12862_2021_1798_MOESM1_ESM.docx]

*BMC Ecology and Evolution*

(Research article — Additional material)

**Molecular and morphological clocks for estimating evolutionary divergence times**

Jose Barba-Montoya^1,2^, Qiqing Tao^1,2^, and Sudhir Kumar^1,2*^

*^1^Institute for Genomics and Evolutionary Medicine, Temple University, Philadelphia, PA*

*^2^Department of Biology, Temple University, Philadelphia, PA*

*^3^Center for Excellence in Genome Medicine and Research, King Abdulaziz University, Jeddah, Saudi Arabia.*

***Corresponding author:**

E-mail: s.kumar@temple.edu

Sudhir Kumar

Temple University

Philadelphia, PA 19122, USA

## Additional figures

**Figure S1.** The linear relationship between internal branch lengths obtained from molecules versus morphology (excluding terminal branches) for (A) Hemiptera, (B) Hymenoptera, and (C) Spermatophyta. The slope, correlation coefficient (*r*) and p-values are shown. The black dashed line represents the best-fit linear regression through the origin. The solid grey line represents equality between estimates.

**Figure S2.** Calibration densities (dark grey bands), 95% HPD-CIs in the time prior (light grey bands), and posterior (colored lines) for 47 nodes in the Hemiptera timetrees under calibration strategies (A) Cr-green, (B) C1-red, and (C) C2-purple. Calibrated nodes are numbered as in Figure 8.

**Figure S3.** Calibration densities (dark grey bands), 95% HPD-CIs in the time prior (light grey bands), and posterior (colored lines) for 55 nodes in the Hymenoptera timetrees under calibration strategies (A) Cr-green, (B) C1-red, and (C) C2-purple. Calibrated nodes are numbered as in Figure 8.

**Figure S4.** Calibration densities (dark grey bands), 95% HPD-CIs in the time prior (light grey bands), and posterior (colored lines) for 17 nodes in the Spermatophyta timetrees under calibration strategies (A) Cr-green, (B) C1-red, and (C) C2-purple. Calibrated nodes are numbered as in Figure 8.

**Figure S5.** The posterior mean times (empty black dots) and 95% HPD-CIs under calibration strategies Cr (green lines), C1 (red lines), and C2 (purple lines) for the molecular subsets are plotted against the combined from Hemiptera, Hymenoptera, and Spermatophyta datasets using linked clock models. The slope, coefficient of determination (*R*^2^) for the linear regression through the origin, and p-values are shown. The black dashed line represents the best-fit linear regression through the origin. The solid grey line represents equality between estimates.
